# Structural Analysis and Study of Gel Properties of Thermally-Induced Soybean Isolate–Potato Protein Gel System

**DOI:** 10.3390/foods11223562

**Published:** 2022-11-09

**Authors:** Fengqiujie Wang, Xuelian Gu, Mingshou Lü, Yuyang Huang, Ying Zhu, Ying Sun, Xiuqing Zhu

**Affiliations:** College of Food Engineering, Harbin University of Commerce, Harbin 150028, China

**Keywords:** soybean protein isolate, potato protein, non-network protein, network backbone, gel properties, protein interactions

## Abstract

Heat-induced composite gel systems consisting of different soybean protein isolate (SPI) and potato protein (PP) mixtures were studied to elucidate their “backbone” and property changes. This was achieved by comparing the ratio of non-network proteins, protein subunit composition, and aggregation of different gel samples. It was revealed that SPI was the “gel network backbone” and PP played the role of “filler” in the SPI-PP composite gel system. Compared with the composite gels at the same ratio, springiness and WHC decrease with PP addition. For hardness, PP addition showed a less linear trend. At the SPI-PP = 2/1 composite gel, hardness was more than doubled, while springiness and WHC did not decrease too much and increased the inter-protein binding. The hydrophobic interactions and electrostatic interactions and hydrogen bonding of the SPI gel system were enhanced. The scanning electron microscopy results showed that the SPI-based gel system was able to form a more compact and compatible gel network. This study demonstrates the use of PP as a potential filler that can effectively improve the gelling properties of SPI, thus providing a theoretical basis for the study of functional plant protein foods.

## 1. Introduction

Gels can be used as carriers of functional ingredients which makes them important to food processing [1]. In recent years, the gelation and modification of plant proteins to obtain better protein network structure and gel properties has become a trending research topic in the food processing industry. The mechanism of plant protein gelation is that heating gradually denatures the protein, exposing more internal hydrophobic groups to the polar environment and undergoing polymerization and linkage behavior [2,3]. The latter is achieved through constant temperature and cooling stages, which enables the protein molecules to further increase their mutual binding force and undergo aggregation arrangement, eventually forming an irreversible three-dimensional gel-protein network structure [4]. The key factors for gel formation are thermal denaturation temperature, pH, heat treatment time, protein composition, and protein concentration [5,6].

Soybean protein isolate (SPI) is one of the important raw materials for the production of food gels. However, SPI alone is not sufficient to obtain good gelation properties and is often used in combination with other ingredients to obtain the desired gel product [7]. Potato protein (PP) has a nutritional value similar to that of milk protein due to its superior quality to most vegetable proteins, and its high solubility and lysine content [8,9]. These properties position it as a potential emerging high-quality protein for the future [10]. Both SP and PP are spherical proteins that can form thermally-induced gels by heat treatment [11,12]. Few studies have focused on the application of soybean isolate and potato proteins in the preparation of composite gels, and only limited information elucidates that the gel aggregation behaviors of SPI and PP are different [13,14]. Furthermore, PP shows stronger thermally-induced gelling properties than SPI based on rheological properties [15,16]. To provide useful guidance to the industry when improving plant protein gels, it is necessary to understand the difference in performance between SPI and PP complexes in thermally-induced gel formation.

Research on improving the gel properties of plant proteins has focused on physical modification of gels, composite protein interactions, and changes in the gel network structure [17]. A recent study exploring the mechanism of composite gel formation with rice proteins and SPI revealed that protein-based additive components can further enhance the potential applications of plant proteins [18]. Enhancing the aggregation of proteins can promote increase in gel strength [19]. However, improved composite gel properties can also be analyzed in terms of gel network proteins and non-network proteins. The structure of SP gels was previously reported to be subdivided into network protein and non-network protein structures [20]. The non-network proteins in soy protein gels affect the energy storage modulus of the gels [21]. In addition, non-network protein components can be present in the gel network as soluble fillers, and the gel network becomes more compact as the concentration of proteins involved in the gel network increases [22]. The current effect on the formation of complex gel systems of SPI and PP under thermal treatment conditions is not clear. If the addition of small molecule PP with lower molecular weight than SPI is used to fill the network when the SPI gel forms, the structural backbone of the network protein, the gel properties can be effectively improved.

Therefore, in this study, the impact of varied SPI/PP contents on the creation of network skeletal structure and gel properties of the composite gel system were examined. Non-network protein ratio, protein electrophoresis, and turbidity were determined firstly, followed by texture, WHC, and rheological properties. The formation mechanisms of the gel system and the corresponding contributions of the two proteins to the gel structure were investigated using protein secondary and tertiary structure, intermolecular forces, and microstructure analyses. This study will help to understand the interactions and properties between SPI and PP, to improve gel properties by modulating the composite gel structure, and to present a new method for SPI-PP gel system applications in novel food productions.

## 2. Materials and Methods

### 2.1. Materials

Low-temperature defatted soybean meal was supplied by Harbin HighTech Group (Harbin, China). Fresh potatoes were obtained from a public store (Harbin, China). Tianjin Tianli Chemical Reagent Co. supplied analytical reagents (AR).

### 2.2. Preparation of Protein and Gel

#### 2.2.1. Preparation of SPI

SPI was prepared as described previously by Wan et al. [23], with slight modifications. The low-temperature defatted soybean meal was ground with a material grinder, and the powder was sieved to ensure that the particle size was lower than 178 μm. The solvent was agitated for 2 h after deionized water was added with 10 times the weight of soybean meal. With 2 M NaOH, the solvent pH was adjusted to 9 and centrifuged at 4000 rpm for 30 min. The protein precipitate was then obtained after the solvent was adjusted to pH 4.5 by 2 M HCl and centrifuged for 30 min at 4000 rpm (25 °C), pH adjustment to neutral, and SPI was obtained after the sample was freeze-dried at −62 °C for 53 h, with a protein content of 92.86 ± 0.02% (*w*/*w*) determined by the Kjeldahl method.

#### 2.2.2. Preparation of PP

PP was as described by Zhao et al. [24], with minor modifications. Fresh potatoes were peeled and crushed to obtain a homogenate. Sodium bisulfite solution (0.1%) with 2 times the weight of the homogenate was added and ground. The mixture was filtered using an 80-mesh filter cloth and centrifuged for 30 min at 10,000 rpm. The next stages were the same as the SPI described earlier and then filtered with a 10 kDa molecular weight filter membrane. The final PP obtained was measured to have 91.5% ± 0.02% protein content (*w*/*w*) using the Kjeldahl method.

#### 2.2.3. Gel Preparation

Heat-induced gels were produced according to that described by Wu et al. [22], with minor modifications. Composite gel solutions with SPI or PP mass of 18 g/100 mL (composite gels prepared at this protein concentration are convenient to handle) in a 100 mL beaker and neutral pH were prepared with deionized water, including five groups with different SPI and PP at ratios of 3/0, 2/1, 1/1, 1/2, 0/3 (*w*/*w*, dry basis). Stirring at 25 °C for 2 h, the protein mixture was fully hydrated. To fully denature SPI-PP, it was heated in a water bath for 30 min at 95 °C to make the gel system, which was kept at 4 °C. After 24 h storage (at 4 °C), parts of the gels were taken to detect gel strength, WHC, and protein network separation, while the rest of the gels were freeze-dried and ground to 80-mesh sieved powder after being pre-frozen overnight at −20 °C. Experimental analyses were performed at 20 °C unless other methods had specific explanations.

### 2.3. Non-Network Protein Ratio (Rnon)

Purification of network and non-network proteins of the gels was determined using the approach described by Wu et al. [22]. The sample gel (20 g) was broken into 1~5 mm pieces and added (10 times the weight of the reagent) to neutral phosphate buffer (0.01 mol/L) along with 0.06 g NaN_3_ (to prevent microbial contamination) in a sample bottle. The bottle and its contents were shaken at 60 rpm for 54 h under ambient condition (25 °C) to ensure that the non-network proteins of the heat-induced composite gel were fully diffused. The buffer solution was aspirated with a polymer polyphenylene syringe and filtered through a 0.22 μm filter followed by membrane filtering. The non-network protein was defined as the soluble protein content in the buffer as assessed by the BCA technique, while the network protein was defined as the residual protein. Rnon was calculated according to Equation (1)

(1) $$\mathrm{Rnon}\;(\%)=\frac{C_{1}\;\times \;V_{1}}{\;C_{2}\;\times \;M_{1}}\times 100\%$$

where C_1_ is the soluble protein content (mg/mL); V_1_ is the phosphate buffer volume; C_2_ is the amount of protein in 1 g of protein gel (g); and M_1_ is the protein gel’s mass (g).

### 2.4. Electrophoresis

Sodium dodecyl sulfate polyacrylamide gel electrophoresis (SDS-PAGE) was conducted using the method of Kobayashi et al. [25] to assess the variations in protein distribution across gel samples. After the non-network protein and the network protein obtained in the previous step were freeze-dried and sieved through an 80 micron-mesh sieve, the electrophoresis samples were prepared together with the freeze-dried powder of the unseparated gel sample. Neutral phosphate solution (0.02 mol/L) of 2 mg/mL was prepared and the 0.5 mL sample solution was mixed with 0.05 mL 5% 2-Hydroxy-1-ethanethiol, 0.2 mL 20% SDS, and 0.25 mL deionzsed water. The sample solution was heated in a boiling water bath for 5 min. Subsequently, the 10 μL sample solution was placed into each well (with 5% stacking gel and 12% separating gel) and subjected to SDS-PAGE (run at 80 V for 30 min and at 120 V for 85 min).

### 2.5. Turbidity Measurement

The turbidity of network and non-network proteins separated by free diffusion was measured. Each sample solution (1 mg/mL) was prepared and absorbance values at 600 nm represented the turbidity of the samples by using an ALPHA 1650 UV-visible spectrophotometer (Shanghai Spectrum Instruments Co., Ltd., Shanghai, China).

### 2.6. Texture Analysis

The texture profile analyses of the composite gel samples were determined by altering the procedure according to Feng et al. [26]. Each gel sample was cut into 2 cm × 2 cm × 2 cm cubes and tested with a texture analyzer, TA new plus (ISENSO Corporation, New York, NY, USA) with a P/0.5 probe. The compression ratio was 35% of the gel sample’s height, and triple parallel tests were conducted on each sample.

### 2.7. Water Holding Capacity (WHC)

WHC of the gel samples was measured based on the method of Zhang et al. [27]. A certain amount of gel samples was weighed and centrifuged (8000 rpm) for 13 min. Water was recovered from the supernatant using absorbent paper, and WHC was determined using Equation (2)

(2) $$\mathrm{WHC}\;(\%)=\frac{m_{2}}{m_{1}}\;\times \;100\%$$

where m_1_ was the starting weight of the gel and the ending weight of the gel after centrifugation was represented by m_2_.

### 2.8. Dynamic Rheology

To expose the rheological features of heat-induced composite gels, frequency scanning of dynamic rheology research was carried out. According to the method described by Katzav et al. [15], each composite gel sample was measured by an MCR 102 advanced rotational rheometer (Anton Paar Shanghai Trading Co., Ltd., Shanghai, China) fitted with a 40 mm clamp. After oscillatory strain scanning at 1.0 Hz and an adjustable angular frequency of 0.1 to 100 rad/s, the strain was measured to be 0.5%, which was within the linear viscoelastic region of the gels. The edges of the plate were covered with liquid paraffin after loading and covering the plate to prevent evaporation of the samples during the test.

### 2.9. Fourier Transform Infrared Spectrum (FT-IR)

FT-IR was utilized to examine each sample protein’s secondary structure, which was achieved by a Spectrum Two infrared spectrometer (PerkinElmer, Waltham, MA, USA) based on the method described by Dong et al. [28]. In this experiment, composite gel samples (3.5 mg) were mixed with KBr (350 mg) powder, tabled, and scanned. The raw spectra were acquired across (4000–400 cm^−1^) and subjected to 32 scans at 4 cm^−1^. PeakFit version 4.12 software and a Gaussian peak fitting algorithm were used to analyze the protein secondary structure. According to the position of the second derivative band distribution of the protein secondary structure described by Wang et al. [29], amide I band in the spectral region (1600–1700 cm^−1^) was selected and the relative relationship of α-helix, β-sheet, β-turn, and random coil percentage was calculated.

### 2.10. Measurement of UV Spectrum

An appropriate amount of gel sample powder was weighed, dissolved with neutral phosphate buffer (0.2 mol/L), and configured into a sample solution of 1 mg/mL. The UV absorption spectrum was measured by a Lambda 365 UV spectrometer (Perkin Elmer Co., Ltd., Waltham, MA, USA) in the range of 190–400 nm.

### 2.11. Intermolecular Forces

The intermolecular forces of heat-induced composite gels were analyzed using the method described by Tanger et al. and Tanger et al. [30,31] with some modifications. In blocking the different forces between gel protein molecules by different chemical reagents as a principle, the following was done. Firstly, each composite gel was crushed to enlarge the sample exposing area for ease of reactions with different reagents. Then, 20 g of the relevant buffer solution (details on buffer B1-B3 formulation can be found in [30] cited above) was added to 0.5 g of each sample in a test tube. After 16 h of shaking (ensuring adequate reaction of gel samples with reagents), the sample was centrifuged (11,000 rpm) for 15 min. The Dumas technique was used to determine the amount of soluble nitrogen in the supernatant. The ratio of soluble nitrogen content of gel samples in different buffers to the soluble nitrogen content contained in the gel samples was calculated. The calculated results were named H1, H2, and H3 for buffer B1, buffer B2, and buffer B3. According to the rules of calculating interaction forces, electrostatic interaction and hydrogen bond was represented by H1, hydrophobic interaction was calculated by (H2 − H1), and disulfide bond was calculated by (H3 − H2), hence the protein interaction forces of the composite gel samples were obtained.

### 2.12. Scanning Electron Microscopy (SEM)

SEM was used to examine the microstructure of heat-induced composite gel samples. Each sample was sliced into 2 × 5 mm (W × L) and fixed in 5 mL glutaraldehyde (2.5% concentration; pH 7.2). Each sample was dehydrated in ethanol and soaked in pure tert-butanol followed by freeze drying. Following gold sputtering, each sample was examined under SEM (Hitachi S-3400N, Tokyo, Japan).

### 2.13. Statistical Analysis of Data

All of the tests were done in triplicate under identical conditions and data were presented as mean ± standard deviation. Statistical significance between all data was tested using analysis of variance (ANOVA) by using SPSS 20.0 software (IBM Corp, Armonk, NY, USA). The Tukey test was used to test for significant differences in the data at a level of (*p* < 0.05).

## 3. Results and Discussion

### 3.1. Gel Structure Analyses

#### 3.1.1. Rnon Analysis

**Figure 1:**
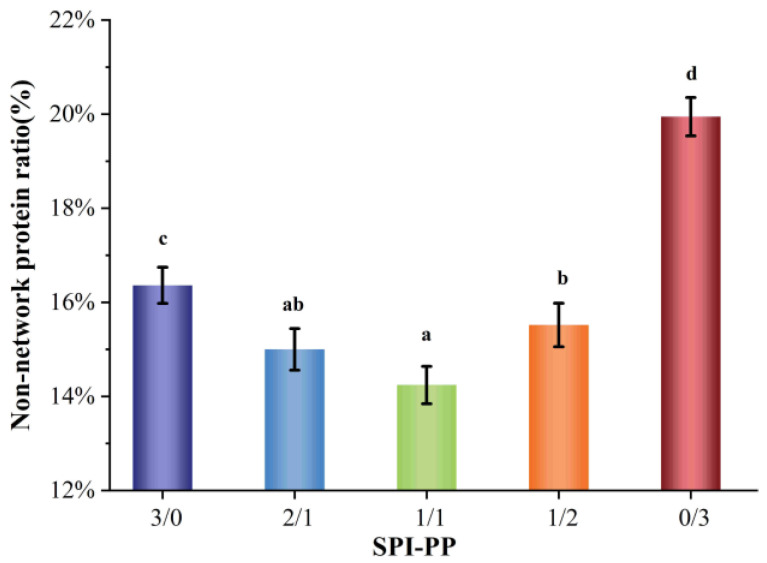
Non-network protein content of composite gels at different SPI and PP ratios. Different lowercase letters indicate significant differences between samples in the same group (*p* < 0.05).

Figure 1 shows the ratio of non-network proteins in a composite gel consisting of SPI and PP. The non-network protein content was lower in the composite gel system when SPI content was dominant, while it gradually increased when PP content was dominant in the system. This difference was by the interaction change between SPI and PP. It was reported that main contributors to the gel network were associated with large molecular weight proteins, such as SPI, which is mainly composed of 7S (150~200 kDa) and 11S (350 kDa) [32]. As a main component of PP, patatin (45~50 kDa) can be partially released during the formation of heat-induced gel [15,33]. Comparing the single SPI gel and single PP gel, the non-network protein composition of a single SPI gel was lower than that of a single PP gel, indicating that SPI was more capable of forming stable macromolecular aggregates than PP. A total protein concentration of 18% for all the composite gel systems was adopted in this study. In addition, due to the high protein concentration of the composite gel itself, undenatured proteins may be present during the heat treatment process and these undenatured proteins may be contained in the isolated non-network proteins [22,34].

#### 3.1.2. Protein Electrophoresis and Turbidity Analysis

**Figure 2:**
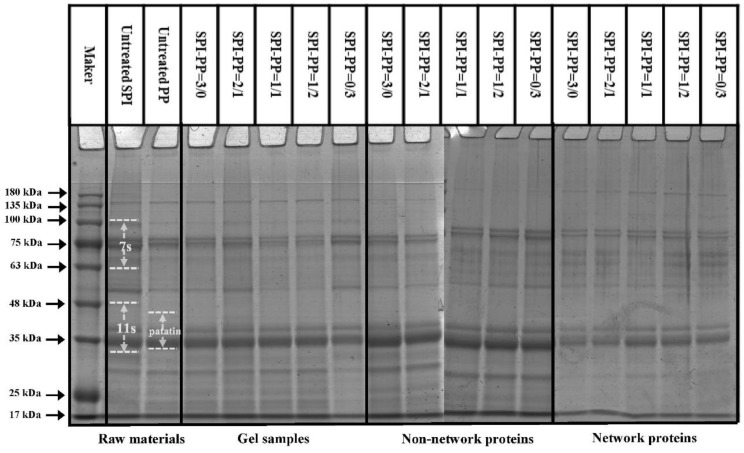
SDS-PAGE analysis of controls and composite gels, non-network proteins, and network proteins at different SPI and PP ratios.

**Figure 3:**
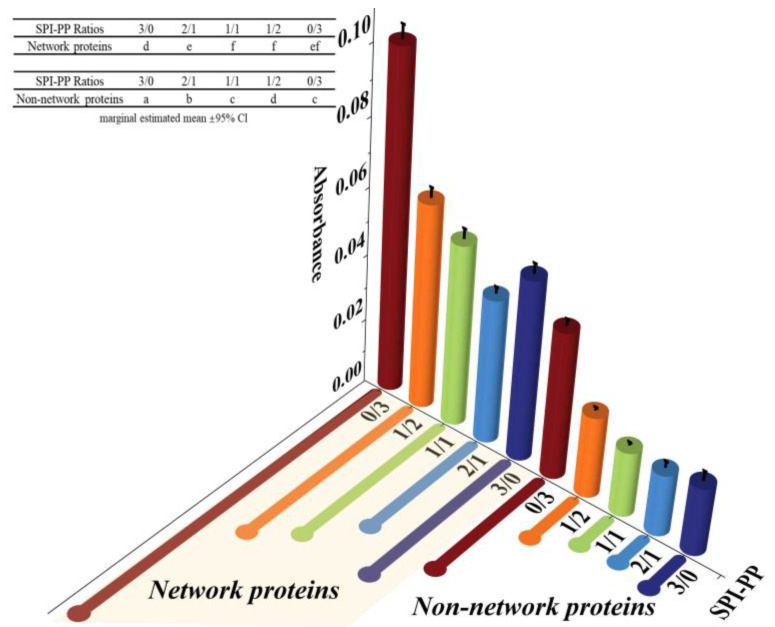
Turbidity analysis of network proteins and non-network proteins at different SPI and PP ratios.

The protein aggregation situations for different composite gel samples were analyzed by SDS-PAGE, which elicited the composition of network and non-network proteins. As illustrated in Figure 2, SPI was dominated by 7S (≈63~100 kDa) and 11S (≈35~48 kDa) subunits, while PP had a small amount of high molecular weight proteins (about 75~100 kDa), large amount of patatin (≈39~43 kDa), and protease inhibitor of low molecular weight (≈5~25 kDa) [35]. In the composite gels formed with different SPI and PP contents, a reduction of the above-mentioned five characteristic bands was observed. New subunit bands at (about 48~75 kDa) and (about 100~135 kDa) appeared, indicating that a cross-linking reaction and aggregation happened after heat treatment of SPI and PP. The darkest electrophoretic bands appeared in the SPI-PP = 2/1 composite gel, indicating a strong cross-linking reaction between SPI and PP. It was shown that after the denaturation of 11S, the main component of SPI during heat treatment easily dissociated from the basic polypeptide that could form the hydrophobic core of the gel protein by hydrophobic interactions and thus aggregated with other components [36]. It was further observed that all composite gels retained characteristic subunit bands of SPI, but the presence of the characteristic bands of PP was weaker. This implies that SPI was more competitive than PP, and this might be evidence that SPI acted as the main gel backbone in the composite gel system. Wu et al. [21] reported electrophoresis results, showing that similar SPI bands were still present in the gel sample after the SPI had been heat treated to form a gel. Similar results were reported by Katzav et al. [15] for potato protein gels prepared at 10% concentration, where the electrophoretic bands were somewhat similar to those of the raw material. It was reported that in the whey protein and PP composite gel system, whey protein, which contains abundant disulfide bonds, was the dominant component. As a larger agglomerate, it could form a more stable gel network structure with low cross-linked patatin [37]. Electrophoresis results of network proteins and non-network proteins of the samples showed that non-network proteins were based on PP characteristic subunit bands. Combining the results with the non-network protein ratio further indicated that the main component of the non-network protein in the composite gel was PP. The network protein bands showed the production of large molecular weight subunit bands at ≈135~180 kDa, signifying that the composite gel used SPI as the gel network backbone. On the basis of which small molecule PP components were cross-linked and filled with it, thus complexed in the SPI gel network as soluble substances constituting the composite protein gel [20]. Turbidity changes can reflect the degree of protein aggregation at the macroscopic level [36]. The turbidity of the non-network proteins with the SPI-PP = 2/1 composite gel was the lowest among all samples, indicating a tight gel network was formed (Figure 3). Turbidity gradually increased with increasing PP concentration, indicating the formation of more insoluble aggregates. The turbidity of network proteins of different protein gel contents above that of non-network proteins was consistent with the electrophoresis results, indicating that network proteins consisted of larger protein aggregates. The above electrophoresis and turbidity results illustrated that PP had a substantial influence on gel formation from SPI aggregates. The formation of soluble aggregates was better with the SPI-PP = 2/1 composite gel, where PP possibly filled the SPI gel skeleton as a soluble material.

### 3.2. Characterization of Gel Properties

#### 3.2.1. Texture Analysis

Texture characteristics are an important index for evaluating protein 3D dimensional structures. Hardness reflects the maximum force required to deform the gel or the strength of the gel network, while elasticity reflects the gel’s ability to recover from stress [38]. Table 1 demonstrated that there was a high degree of variability in the textural properties of gels with different SPI and PP contents, indicating that PP and SPI contents had a significant influence on gel textural properties. The gel hardness formed by PP alone was stronger than that formed by SPI alone, but elasticity showed an opposite trend, indicating that each of the two proteins had its qualitative advantages. When the system was constituted by the SPI-PP = 2/1 composite gel, gel yielded good hardness, meaning closer cross-linking behavior occured. This was probably due to PP acting as filling material in the SPI heat-induced polymerized protein backbone to form a compact composite gel network. Hardness decreased when the SPI-PP = 1/1 composite gel, proving that the main structure was sustained by SPI and proper addition of PP increased the mechanical strength of the composite gel. Conversely, as PP self-aggregates increased in the composite gel, hardness significantly increased (the SPI-PP composite gel maximum ratio was 1/2), while the PP amount in the SPI protein skeleton decreased. This was because low thermal denaturation temperature protein produced excessive denatured proteins by heat treatment, leading to stronger aggregations [39]. Interestingly, the elasticity decreased with increase in PP content, implying that PP limited the gel elasticity. Therefore, the addition of PP had better utility in improving the hardness of the composite gels compared to SPI, but too much addition was detrimental to the elasticity of composite gels.

#### 3.2.2. WHC Analysis

WHC is an important index of composite gel quality formed by protein aggregates and reflects the water holding ability of gel components (2). WHC of SPI was higher than PP (Table 1.). In the composite gel system, the WHC of the SPI-PP = 2/1 composite gel was similar to the single SPI, while gradually decreasing as the PP ratio increased from 1/2 to 2/1. Over addition of PP could not trap water in the gel system, resulting in the cross-linking difference between SPI and PP, which might be related to the interaction force between SPI and PP [40]. Additionally, random aggregation behavior of globular proteins occurred when their thermal denaturation temperature was too high, causing the WHC to be defective due to a rougher interior structure of the gel network [41]. The higher WHC represented more retainment of water content in the gel system, leading to an increase in gel elasticity [42].

#### 3.2.3. Rheological Properties Analysis

**Figure 4:**
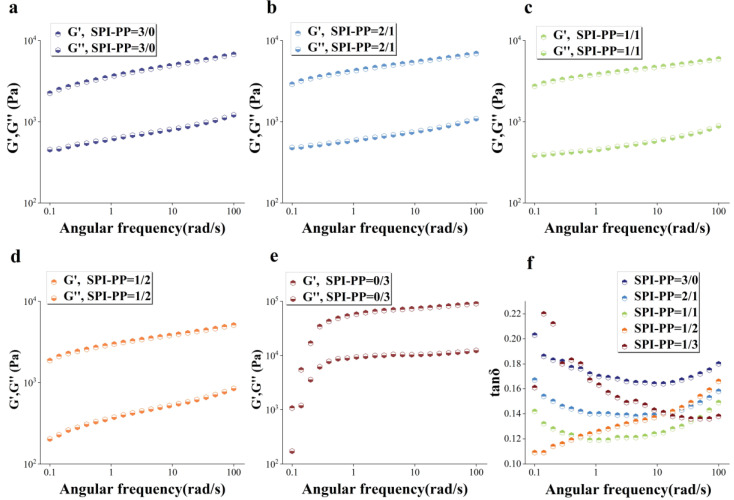
Dynamic rheological diagrams of composite gels. (**a**), G′, G″(**a**–**e**) of SPI-PP = 3/0; (**b**) SPI-PP = 2/1; (**c**) SPI-PP = 1/1; (**d**) SPI-PP = 1/2; (**e**) SPI-PP = 0/3; and (**f**) tanδ of composite gels.

The gel elastic properties for different PP and SPI contents under frequency sweep condition was studied (Figure 4). The solid-like qualities were connected to the storage modulus (G′) value as a specific representation of gel hardness, while the loss modulus (G″) value was related to the liquid-like properties [43]. Among them, G′ is the best indicator of gel structure formation, and G′ usually shows an increasing trend in line with gel network formation, suggesting that the gel’s mechanical characteristics are gradually improved [44]. For every sample, G′ was greater than G″ in Figure 4a–e, indicating that the sample gel network structure was continuously dominated by elasticity. There was a distinct difference in the single PP sample compared to other test groups. The PP sample had the highest G′ values in accordance with the previously described gel hardness, due to its lower thermal denaturation temperature than SPI, and certain degree of protein denaturation significantly enhanced its gel network force [3,45,46]. While in the composite gel system, the G′ value of the SPI-PP = 2/1 composite gel increased, but gradually decreased with decrease in SPI content. This phenomenon was ascribed to the state of protein differentiation and aggregation induced by both SPI and PP, revealing that SPI was dominant in the composite system and constituted the main gel network frame. Lv et al. [47] showed that the addition of PP enhanced the G’ and G” of SPI gels. The gel aggregation degree of spherical proteins by heat treatment led to changes in the gel storage modulus [48]. In addition, it was reported that PP has only a trace number of sulfur-containing groups, and the addition of exogenous sulfur-containing components had a catalytic effect on its gelation [24]. When PP content was low, it possibly acted as a filler in the gel network, thereby producing better cross-linking effects among the composite gel protein molecules. SPI enhanced the gel network by strengthening the disulfide bonds or hydrogen bonding and increased the G′ of the composite gel.

However, when PP content increased, the covalent bonding energy that mainly supported the maintenance of the composite gel decreased and transformed into hydrophobic interaction forces with lower bonding energy as the supporting force of the composite gel, leading to the reduction of the gel system. This will be explained in detail in Section 3.3.3. This phenomenon might also be related to the decrease in gel WHC with increasing PP content as mentioned above. The loss of water inside the gel’s 3D network interspaces resulted in a decrease in gel elasticity. Excessive self-aggregation in the single PP system was induced by heat treatment. This was revealed by the enhanced folding of the protein secondary structure and the higher hydrophobic interaction, and thus formed a stronger self-supporting heat-induced gel [49]. The loss modulus (tanδ; G″/G′) reflects the rheological changes due to the intermolecular interactions of the sample and represents whether the gel is in the solid state (tanδ < 1) or liquid state (tanδ > 1) [50]. Figure 4d shows that all gel samples had tanδ values less than 1 representing solid gels, and their gel behaved more elastic rather than viscous. Similarly, the elastic properties of the composite gels of SP and cod protein were further strengthened as the cod quantity increased [51]. The elasticity of the composite gel decreased when SPI content was lower than PP, while when it was higher than PP, tanδ value changes were not evident according to angular frequency changes ranging from 0.139 to 0.158. This indicated that the composite gel was more stable under this condition, owing to the 3D gel network formed by SPI dominance and PP filling interactions.

### 3.3. Protein Molecular Structure of Gels

#### 3.3.1. Fourier Infrared Spectroscopic Analysis

**Figure 5:**
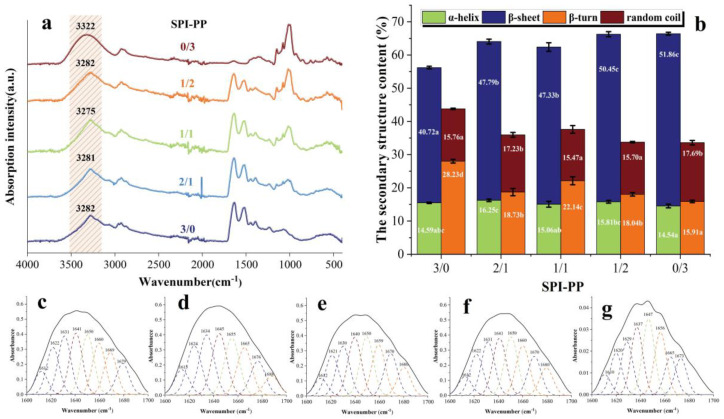
FT−IR spectra of gel samples. (**a**) Secondary structure content of composite gels at different SPI and PP ratios; (**b**), the fitting peaks of the infrared spectra of gels (1700−1600 cm^−1^); (**c**) SPI-PP = 3/0; (**d**) SPI-PP = 2/1; (**e**) SPI-PP = 1/1; (**f**) SPI-PP = 1/2; and (**g**) SPI-PP = 0/3. Different lowercase letters in the figure indicate significant differences between samples in the same group (*p* < 0.05).

In FT-IR spectra, the secondary structure of the protein is often expressed by the amide I band (1700–1600 cm^−1^), and the relationship between the amount of α-helix, β-sheet, β-turn, and random coil is characterized by fitting the absorption peak size [29]. The absorption peaks of SPI-PP samples at (1700–1600 cm^−1^) IR fitted fractions are shown in Figure 5a, and secondary structure contents are shown in Figure 5b. β-sheet reflects the rigid structural strength of the gels. The highest β-sheet content was found in the SPI-PP = 0/3 composite gel, which was 27.36% higher than that of the SPI-PP = 3/0 composite gel, indicating that the structure of the latter was more ordered with better stability [52]. This finding was in accordance with the qualitative properties (hardness) previously described. In the composite gels, the β-sheet and random coil contents tended to increase as the PP content increased. Whereas, α-helix and β-turn angle contents were opposite, resulting in a decrease in gel elasticity, which was consistent with the above-mentioned texture results, suggesting that PP changed the interaction of the composite gels. In addition, there was a vibrational transformation of -OH in the range of (3600–3100 cm^−1^) [53]. Note that this zone contains only the SPI group at a lower wave number (3282 cm^−1^). However, the hydrogen bonding interaction of the composite gel changed with different PP and SPI contents. At the SPI-PP = 2/1 composite gel, its α-helix content increased, which indicates enhancement of hydrogen bonding within the two protein molecules [54]. As the SPI-PP ratio increased to 1/1, the absorbance shifted to lower wavelengths of 3281 and 3275 cm^−1^, which confirmed that addition of PP enhanced the strength of intermolecular hydrogen bonds in the composite gel system. On the other hand, when the composite gel system was dominated by PP, hydrogen bonding interactions weakened, as reflected by the increase in wavelength to 3282 and 3322 cm^−1^. In addition, in Figure 5a, the wavelengths of 2929–2923 cm^−1^ indicated the alteration of the -CH group and -NH^3+^ of the samples [55]. Compared with PP, peaks of the composite gels gradually shifted to higher wavelengths, proving that the intermolecular interactions between PP and SPI increased with decreasing PP content, and SPI acted as the main frame of the composite gel system.

#### 3.3.2. UV-Visible Spectra Analysis

**Figure 6:**
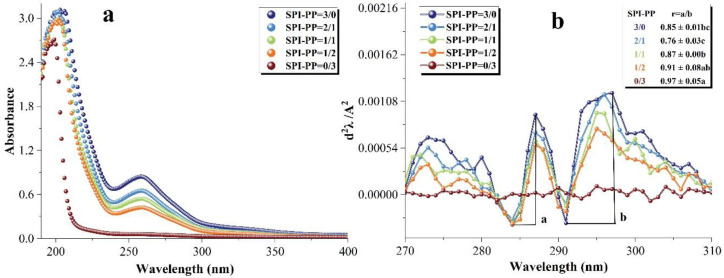
Changes in the UV–visible spectra of composite gels at 190–400 nm. (**a**) Second-order derivative UV–visible spectra and (**b**) corresponding values of r = a/b at (280–300 nm). Different lowercase letters in the figure indicate significant differences between samples in the same group (*p* < 0.05).

UV-visible spectroscopy is used to detect the properties of protein side chains containing aromatic amino acids that can absorb UV-visible light and produce specific absorption peaks, which can reflect changes in the tertiary structure of the protein [56]. Figure 6a shows the UV-visible spectra of the samples. The red or blue shift of these peaks reveal a shift in the protein structure towards polarity or non-polarity. There was a maximum absorption peak near the initial UV wavelength of 200 nm, which was due to π-π * variation of the C=O double bond contained in the protein [57]. In the composite gels containing SPI, the peak shifted towards blue as the percentage of PP increased, indicating that SPI-PP underwent inter-binding interaction, leading to a shift of the protein microenvironment of both toward non-polarity. After the second derivative processing of the spectrum, the peak and trough distances (ratio of a to b) were calculated for the presence of two pairs of positive and negative absorption peaks in the range of 280–300 nm to obtain the r-value, which was positively correlated with the degree of tyrosine and tryptophan exposure [56,58,59]. As shown in Figure 6b, PP exposed more tertiary structures during gel formation, i.e., the greatest degree of tyrosine and tryptophan exposure was within a single PP protein. In the SPI and PP composite gel system, the lowest r value was observed when the percentage of SPI was larger than that of PP, indicating that the folding of the composite gel structure at this time allowed more aromatic residues to be encapsulated in the protein molecule. This may be due to PP exposing its protein tertiary structure under heat treatment to maintain better SPI interactions with the protein, thus forming a composite gel as a filler of the SPI network gel [60].

#### 3.3.3. Intermolecular Forces Analysis

**Figure 7:**
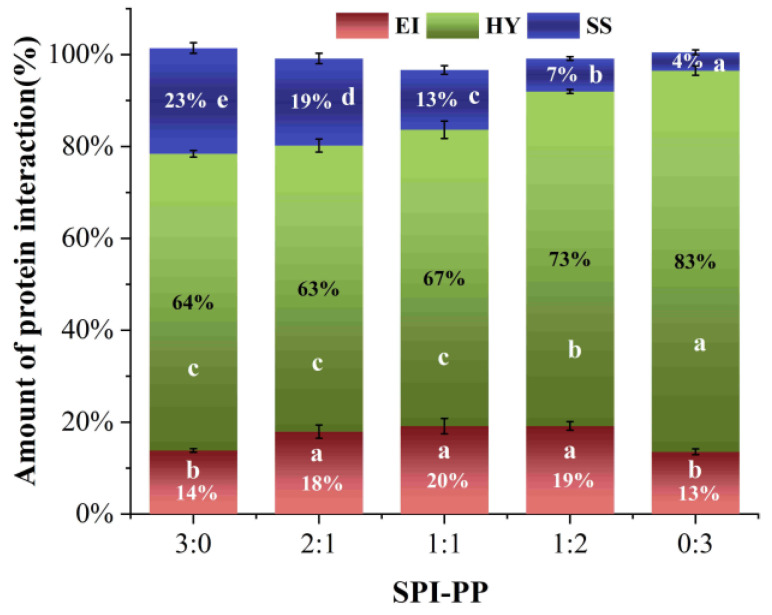
Molecular interaction force analysis of composite gels at different SPI and PP ratios (EI: electrostatic interaction and hydrogen bond; HY: hydrophobic interaction; SS: disulfide bond.). Different lowercase letters in the figure indicate significant differences between samples in the same group (*p* < 0.05).

Differences in protein molecular structures led to different gel characteristics, and electrostatic interactions, hydrogen bonds, hydrophobic interactions, and disulfide bonds greatly influenced protein molecular binding forces [61]. It was reported that heat-induced SPI gels are physical gels dominated by non-covalent interactions [62]. Hydrophobic interactions are the forces that exist between non-polar groups of proteins. As shown in Figure 7, the major factors in the production of protein gels during heating were hydrophobic interactions, and the relative content of hydrophobic interactions in the gel system exhibited a continuous increase with increase in PP [37]. Ionic and hydrogen bonds are commonly detected in the polar groups of protein molecules [63]. The heat treatment broke the hydrogen bonds, which maintained protein conformations, changed the electrostatic interactions, unfolded molecular chains, and exposed more internal groups. During the cooling and standing process at 4 °C, parts of the denatured proteins were restored, thereby hiding a certain hydrophobic group. While, charged polar groups recombined with each other to form new ionic and hydrogen bonds which stabilized the gel conformation, thus the content was maintained at the similar level [6]. Disulfide bonds are strong covalent bonds formed between protein peptide chains after oxidation of cysteine residues, which are vital in maintaining the spatial structure of the 3D gel network [37]. SPI contained more sulfur groups than PP [23]. The disulfide bonds formed inside the protein molecules were significantly lower when the PP content was dominant in the gel systems, which explained that disulfide bonds only accounted for 4.00% in the SPI-PP = 0/3 composite gel. The aforesaid findings showed that during the development of heat-induced gels, covalent and non-covalent cross-linking occurred between distinct SPI and PP contents. Among which, the SPI-PP = 0/3 composite gel had a strong covalent cross-linking phenomenon. The electrostatic interaction forces and hydrogen bonds between the protein molecules maintaining polar groups were also stable. This was consistent with the second-order derivative UV spectroscopy results.

#### 3.3.4. Microstructure Analysis

**Figure 8:**
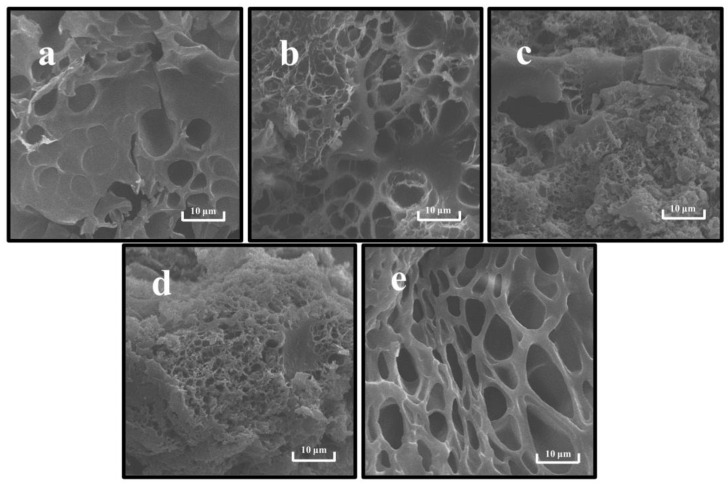
Scanning electron microscopy of composite gels at different SPI and PP ratios. (**a**) SPI-PP = 3/0; (**b**) SPI-PP = 2/1; (**c**) SPI-PP = 1/1; (**d**) SPI-PP = 1/2; and (**e**) SPI-PP = 0/3. Magnification is 5000×.

SEM can characterize the microstructures and component interactions of gels [18]. As shown in Figure 8, the gel network formed by a single PP was the loosest with coarse pores, while the gel network formed by a single SPI was relatively tight and fewer pores appeared in the cross-section. This indicates that the gel skeleton morphology formed by SPI and PP differed significantly. Correlating the network pores with the WHC results, it was found that the rough network pores affected and reduced the gel WHC, which concurs with findings of prior studies [27]. In the composite gel system, the interaction between PP and SPI protein particles was observed, thus changing the gel network structure. When the SPI content was predominant, a more homogeneous network retained the cross-section, thereby increasing the degree of cross-linking. Additionally, the compatibility between PP and SPI was higher, leading to the formation of a complex dominated with SPI having a “gel network skeleton” and “filled” with PP by cross-linking in a 3D gel network. In contrast, in Figure 8c,d, the content of disulfide bonds that sustained the 3D structure of the gel was reduced due to a decrease in SPI, which provided covalent interaction. This enabled it to gradually lose its function as the dominant gel network [19]. On the other hand, irregular aggregation of the two proteins occurred, thereby forming hydrophobic bonds with lower bond energy that were unable to hold part of the water in the gel system [14]. The reduction of bond energy and water led to the differentiation of the microstructure and the formation of a relatively rough and disordered gel network structure [64].

**Figure 9:**
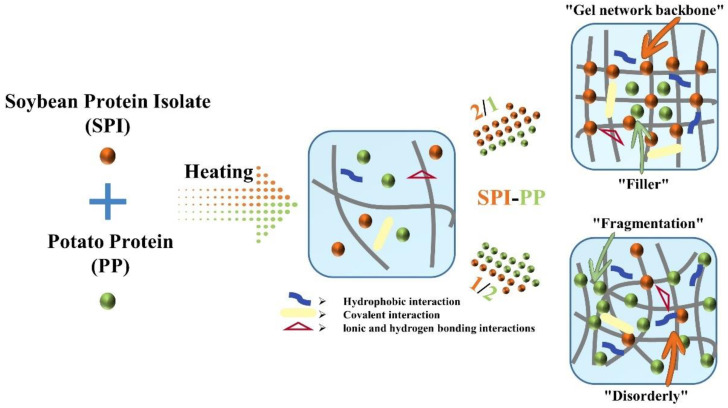
Schematic diagram of thermally-induced composite gel formation prepared from SPI as “gel network backbone” and PP as “filler”.

Based on a comprehensive analysis of heat-induced composite gels prepared from different contents of SPI with PP, we proposed a possible mechanism that provides more perspectives on the structural changes (Figure 9). Initially, PP in a small amount improved the texture properties and non-covalent interactions of SPI gel. Gradually, a more homogeneously distributed gel network structure dominated by SPI was formed. However, the increasing ratio of SPI-PP (from 2/1 to 1/2) resulted in the formation of irregular agglomerates with rough mesh, which affected the porosity and continuity of the structure inside the gel. Hence, the over-added PP would reduce the springiness, water-holding capacity, and storage modulus (namely gel properties) of the samples. The best gel was obtained at the SPI-PP = 2/1 composite gel.

## 4. Conclusions

SPI acted as the skeleton of the composite gel, and PP acts more as a dependent filler. The cross-linking reactions between PP and SPI were established by FT-IR spectra and by the shift in the microenvironment. Optimal PP concentration in the composite gel beneficial in enhancing the composite gel properties was established. β-sheet is a characteristic protein molecular secondary structure of PP, where the aromatic amino acids are located, and indicates that the SPI is cross-linked with PP. Hydrophobic interactions played a major role in the complex gel system, with disulfide bonds playing a minor role, along with electrostatic interaction forces and hydrogen bonds. The different occupancy of SPI and PP changed the bonding forces supporting the gel complex. The SPI-dominated composite gel microstructure produced better protein aggregation behavior and the formation of tight, stable gel networks compared to PP. This study determined the difference between the gel backbone and filler in two different protein composite systems by taking the change of protein molecular subunits and the perspective of non-network proteins, and to gain insight into the mechanism of the influence of SPI on PP through the molecular properties of proteins and gel properties.

## Figures and Tables

**Table 1:** Hardness, springiness, and WHC of composite gels at different SPI and PP ratios.

SPI/PP (*w*/*w*)	Hardness (N)	Springiness	WHC (%)
3/0	1.53 ± 0.01 ^a^	0.83 ± 0.01 ^d^	98.33 ± 0.01 ^c^
2/1	3.62 ± 0.01 ^c^	0.75 ± 0.01 ^b^	98.30 ± 0.22 ^c^
1/1	3.18 ± 0.02 ^b^	0.67 ± 0.01 ^e^	91.84 ± 0.10 ^b^
1/2	4.45 ± 0.02 ^d^	0.61 ± 0.02 ^a^	91.84 ± 0.89 ^b^
0/3	14.53 ± 0.03 ^e^	0.53 ± 0.02 ^c^	76.42 ± 0.97 ^a^

Note: Different letters indicate significant differences between samples in the same column (*p* < 0.05).

## Data Availability

Data is contained within the article.
